# An inhibitory glycinergic projection from the cochlear nucleus to the lateral superior olive

**DOI:** 10.3389/fncir.2023.1307283

**Published:** 2023-12-01

**Authors:** Dennis J. Weingarten, Eva Sebastian, Jennifer Winkelhoff, Nadine Patschull-Keiner, Alexander U. Fischer, Simon L. Wadle, Eckhard Friauf, Jan J. Hirtz

**Affiliations:** ^1^Animal Physiology Group, Department of Biology, RPTU University of Kaiserslautern-Landau, Kaiserslautern, Germany; ^2^Physiology of Neuronal Networks Group, Department of Biology, RPTU University of Kaiserslautern-Landau, Kaiserslautern, Germany

**Keywords:** mouse, hearing, superior olivary complex, patch-clamp, optogenetics

## Abstract

Auditory brainstem neurons in the lateral superior olive (LSO) receive excitatory input from the ipsilateral cochlear nucleus (CN) and inhibitory transmission from the contralateral CN via the medial nucleus of the trapezoid body (MNTB). This circuit enables sound localization using interaural level differences. Early studies have observed an additional inhibitory input originating from the ipsilateral side. However, many of its details, such as its origin, remained elusive. Employing electrical and optical stimulation of afferents in acute mouse brainstem slices and anatomical tracing, we here describe a glycinergic projection to LSO principal neurons that originates from the ipsilateral CN. This inhibitory synaptic input likely mediates inhibitory sidebands of LSO neurons in response to acoustic stimulation.

## 1 Introduction

The LSO of the superior olivary complex (SOC) in the auditory brainstem is thought to be the main structure analyzing sound source direction based on interaural level differences (Tollin, 2003; Friauf et al., 2019). LSO neurons receive excitatory synaptic input via the ventral acoustic stria (VAS) originating from the ipsilateral CN and tonotopically matched inhibitory input from the contralateral side. The latter is realized by glutamatergic projections from the contralateral CN terminating in the MNTB, whose neurons then send inhibitory, glycinergic projections to the LSO. The output of LSO neurons thus reflects the direction of a sound source relative to the head, with high activity levels reflecting a position on the ipsilateral side (Boudreau and Tsuchitani, 1968). These circuitries have become model systems for studying the development of inhibitory synapses (Kandler and Friauf, 1995; Kim and Kandler, 2003; Cramer, 2005; Noh et al., 2010; Alamilla and Gillespie, 2013; Fischer et al., 2019), integration of excitation and inhibition (Beiderbeck et al., 2018; Gjoni et al., 2018), and tonotopy (Kim and Kandler, 2003; Hirtz et al., 2012).

The classical view of a purely excitatory nature of synaptic LSO inputs from the ipsilateral CN has been challenged by Wu and Kelly (1994) who reported inhibitory synaptic currents *in vitro* in mouse LSO neurons when electrically stimulating the ipsilateral VAS at various sites, or even the CN directly. In fact, inhibitory effects within the cat LSO upon ipsilateral sound stimulation had been postulated already by Brownell et al. (1979). The origin of this inhibitory projection has, however, so far been unexplored. Glendenning et al. (1991) reported glycinergic projections from the cat CN to the ipsilateral SOC using retrograde tracing, yet these projections could not be unequivocally attributed to the LSO. Nevertheless, the experiments suggested the CN as the most likely source of ipsilateral inhibitory LSO input.

By using a combination of electrophysiology, optogenetical and anatomical tracing, we here prove the existence of an inhibitory, glycinergic projection from the mouse CN to the ipsilateral LSO. Our data highlight the complexity of synaptic circuits in the auditory brainstem.

## 2 Materials and methods

### 2.1 Animals

Experiments were performed using C57BL/6 N wild-type and GlyT2-Cre-tdTomato mice. GlyT2-Cre-tdTomato mice were generated by crossing a tdTomato^Rosa26StopFlx^ mouse line (Madisen et al., 2010) obtained from Hongkui Zeng (Allen institute, Seattle, WA, United States) with a GlyT2-Cre-knock-in-line (Foster et al., 2015) obtained from Prof. Hanns Zeilhofer (University Zürich, Zürich, Switzerland). Animals were housed in the animal breeding facility at the RPTU with a day-night cycle of 12 h, food and water *ad libitum*. The study was performed in accordance with the guidelines of the German Animal Welfare Act and the European Directive 2010/63/EU for the protection of animals used for scientific purposes. Animal experiments were approved by the regional council of Rhineland-Palatinate (Landesuntersuchungsamt Rheinland-Pfalz) with the file numbers G11-2-030 (dextran tracer injections) and G19-2-016 (vector injections).

### 2.2 Unilateral anterograde tracer injection

Three adult (P80-85) wild-type mice were intramuscularly anesthetized using a mixture of ketamine hydrochloride (80 mg/kg) and the sedative xylazine (14 mg/kg). They were mounted in a stereotactic apparatus (Modell 900, David Kopf Instruments) and a craniotomy was carried out once they were areflexic. All tracer injections into the CN were identified by stereotactic coordinates obtained from a mouse brain atlas (Franklin and Paxinos, 2008). From interaural midpoint, displacement along rostrocaudal (−1.88 mm), lateral (−2.19 mm) and depth (+0.2 mm) coordinates was performed. Biotinylated dextran amine (10,000 Dalton MW, Invitrogen, 10% in distilled water) was injected iontophoretically (−10 μA, 7 s on / 7 s off) from borosilicate glass capillaries (GB150-8P; Science Products) with tip diameters of 50–55 μm. To abbreviate anaesthesia, atipamezole (1 mg/kg) was injected subcutaneously. Animals were allowed to recover and survive 5 days before transcardial perfusion.

### 2.3 Perfusion and tissue processing

Mice were deeply anesthetized using intraperitoneal injections of chloral hydrate (700 mg/kg) and transcardially perfused with PBS (pH 7.4), followed by 4% (w/v) paraformaldehyde (PFA). Brains were postfixed in 4% PFA for 2 h and kept in 30% sucrose/PBS solution for cryoprotection for at least 24 h. Thereafter, 30-μm-thick coronal brainstem sections were cut with an HM 400R sliding microtome (Microm), collected in 15% sucrose/PBS, and rinsed in PBS.

### 2.4 Immunohistochemistry and tracer labelling

After rinsing in 0.5% Triton X-100/PBS, slices were blocked for 1 h in 3% bovine serum albumin, 10% goat serum, and 0.3% Triton X-100 in PBS, pH 7.4. Streptavidin conjugated with Cy3 (Jackson ImmunoResearch) was added to a final concentration of 1:200 to label the biotinylated dextran. After 90 min incubation, slices were rinsed in PBS and incubated in the primary antibody serum at 4°C overnight [guinea pig anti-glycine transporter 2 (GlyT2; A1773; Millipore), 1:10,000 in carrier solution (0.3% Triton X-100, 1% bovine serum albumin, 1% goat serum in PBS)]. After rinsing in PBS, slices were incubated for 1 h at room temperature with the secondary antibody goat anti-guinea pig conjugated to Alexa Fluor 488 (Invitrogen), diluted 1:1,000. After three rinsing steps, sections were mounted on glass slides and air dried. Home-made mounting medium containing 30% glycerol, 12% polyvinyl alcohol, 0.005% phenol, ~0.05 M TRIS, and 2.5% 1,4-diazabicyclo[2.2.2]octane (DABCO; Sigma-Aldrich) was used to cover the sections.

### 2.5 Adeno-associated viral vector (AAV) injections

To enable specific activation of inhibitory, glycinergic neurons projecting from the CN to the LSO, a double-floxed Channelrhodopsin 2 (ChR2, AAV5-EF1a-double-floxed-hChR2(H134R)-EYFP) was injected into the CN of GlyT2-Cre-tdTomato mice. pAAV-EF1a-double floxed-hChR2(H134R)-EYFP-WPRE-HGHpA was a gift from Karl Deisseroth (Addgene viral prep #20298-AAV5; http://n2t.net/addgene:20298; RRID:Addgene_20298). Injections were performed at P43-46 and electrophysiological recordings were performed 18–20 days after injection. Animals were deeply anesthetized via isoflurane inhalation (5% initial, 1%–3% during procedure). They were placed on a heating mat into a stereotactic frame. Following application of systemic analgesia (5 mg/kg carprofen), removal of hair, disinfection of the skin using Braunol, and local administration of 2% lidocaine, the skin was opened. The location of the CN for injection of AAV vectors was determined using stereotactic coordinates. A small hole was drilled into the skull using a dental drill. 350 nL vector of 7.7 × 10^12^ GC/ml was injected at 80 nL/min at the desired depth using a thin cannula (NanoFil, WPI) and a micropump (UMP3, WPI). Five minutes after the end of the injection, the cannula was withdrawn, and the skin was sutured. Isotonic, body-warm NaCl-solution was administered subcutaneously during the procedure to ensure hydration of the animal. After recovery on a heating mat, the animals were returned to their cage and monitored daily until the preparation of acute slices. Carprofen (5 mg/kg) was administered on the first two days following surgery.

### 2.6 Preparation of acute brain slices

Adult mice were anesthetized in a chamber ventilated with 3–5% isoflurane prior to decapitation, juvenile mice (P10-11) were decapitated without anesthesia. The skull was cut open from caudal to rostral and removed. The brain was lifted using forceps and cranial nerves were carefully cut. The dissected brain was immediately transferred into ice-cold carbogen-bubbled NMDG-HEPES recovery solution for adult mice (in mM: 93 NMDG, 2.5 KCl, 1.2 NaH_2_PO4, 30 NaHCO_3_, 20 HEPES, 25 glucose, 5 L-ascorbic acid, 3 myo-inositol, 3 Na-pyruvate, 93 HCl, 10 MgCl_2_, 0.5 CaCl_2_) or an ice-cold carbogen-bubbled glucose-based solution (in mM: 1.25 NaH_2_PO_4_, 2 Na-pyruvate, 3 myo-inositol, 26 NaHCO_3_, 260 glucose, 6 MgCl_2_, 0.5 CaCl_2_) in case of juveniles. Cerebral cortex, cerebellum, and the spinal cord were removed. The brainstem was caudally glued onto a cutting plate. For experiments involving electrical stimulation of VAS axons, brains were tilted 10–15° off the coronal axis to maintain the integrity of the CN-SOC projection. 270–300-μm-thick coronal slices were cut using a vibratome (VT 1200S, Leica) at 0.03 mm/s speed. Slices containing the desired area were incubated in carbogen-bubbled NMDG-HEPES recovery solution at 37°C for 11 min in case of preparations from adult mice or in aCSF (in mM: 125 NaCl, 2.5 KCl, 1.25 NaH_2_PO_4_, 2 Na-pyruvate, 3 myo-inositol, 0.44 L-ascorbic acid, 25 NaHCO_3_, 10 D-glucose (H_2_O), 1 MgCl_2_, 2 CaCl_2_) at 37°C for 55 min in case of juveniles. Afterwards, slices were kept in carbogen-bubbled aCSF at room temperature.

### 2.7 Electrophysiology

Acute brain slices were transferred to a recording chamber and fixed with a U-shaped platinum-iridium grid with nylon filaments. They were perfused with carbogen-bubbled aCSF using a pump-driven perfusing system (ISM796B, Ismatec). The recording chamber was secured onto an upright microscope (Eclipse E600FN, Nikon, Tokyo, Japan or Axioskop 2 FS, Carl Zeiss). For visualization of neurons, an IRIS 9 (Teledyne Photometrics), Zelux (Thorlabs GmbH), Orca-05G CCD (Hamamatsu Photonics), or Retiga ELECTRO–M-14-C (Q Imaging) camera as well as a low magnification objective (Nikon CFI Achromat, 0.10 NA or Zeiss Fluar 5×/0.25 ∞/0.17) and a 60x objective (Nikon CFI Fluor, 1.00 NA; Nikon NIR Apo, 1.0 NA or Olympus LUMPlanFL N 1.00 NA) were used. A micromanipulator (Luigs & Neumann, Sutter Instruments, or Sensapex) was utilized to control pipette movement. Patch pipettes were pulled from glass capillaries with filaments (GB150F-8P Science Products) using a P-87 horizontal puller (Sutter Instruments) with resistances of 3–8 MΩ. Pipettes were filled with intracellular solution (in mM: 130 K-gluconate, 5 KCl, 2 MgSO_4_, 10 HEPES, 5 EGTA, 7 Na-Phosphocreatine, 2 Na-Pyruvate, 2 ATP-Na_2_, 0.3 GTP-Na_2_ or 140 K-gluconate, 5 EGTA, 1 MgCl_2_, 2 ATP-Na_2_, 0.3 GTP-Na_2_, pH adjusted to 7.3 using KOH, 280–290 mOsmol) and connected to a patch-clamp EPC9 or EPC10 amplifier (HEKA Elektronik GmbH). Series resistance was compensated between 50–80%, and the liquid junction potential was corrected to 18.7 mV or 15.4 mV, depending on the intracellular solution. Recordings were obtained using a low-pass filter between 7.4 to 8.3 kHz and sampled at 20 kHz. Recording protocols were initiated using PatchMaster v2.69 or v2×90.4 (HEKA Elektronik). All experiments were performed on LSO principal neurons identified by an I_H_ (Sterenborg et al., 2010). Physiological temperature (37 ± 1°C) was used in experiments involving electrical stimulation of fibers, whereas experiments using optogenetics were done at room temperature.

### 2.8 Electrical and optical stimulation

To electrically evoke inhibitory postsynaptic currents (IPSCs), a glass electrode with a tip diameter of 5–10 μm was placed on the VAS. To electrically evoke unitary IPSCs, minimal stimulation intensity was used, which likely activates only a single axon (Stevens and Wang, 1994). Test pulses were applied at 1 Hz and the position of the stimulation electrode in the VAS was slightly changed until IPSCs were detected. Stimulation intensity was increased until IPSCs were reliably evoked with a minimal stimulus amplitude. Ten pulses were applied at 1 Hz for normalization (set to 100%). To block glutamatergic transmission, 20 μM CNQX (Abcam) and 50 μM D-AP5 (Abcam) were washed in for 10 min during which 1-Hz stimulation was done. For another 10 min, 0.5 μM of the glycine receptor antagonist strychnine (Sigma Aldrich; Jonas et al., 1998) was added. Finally, the specific GABA_A_ receptor antagonist GABAzine was washed in (Sigma Aldrich, 10 μM). In a separate set of experiments aimed at determining the number of inputs converging onto a single LSO neuron, a bipolar copper electrode was placed on the VAS laterally to the exit of the facial nerve. IPSCs were elicited with 100-μs monopolar pulses (STG 4002; Multi Channel Systems). Excitatory transmission was blocked by bath application of CNQX and D-AP5. Stimulation intensity was gradually increased every 10 stimuli for 50 μA to a maximum of 3,000 μA, comprising a total of 600 stimulus pulses.

For light stimulation, 10-ms pulses of 470 nm wavelength were applied to the whole slice using an LED (M470L3 or M470L5-C5, ThorLabs GmbH) focused through the 60x objective. To control timing and intensity, the analog output of the HEKA amplifier was connected to the LED driver (Thorlabs).

### 2.9 Microscopy

Images were obtained using a confocal LSM 700 laser scanning microscope (Carl Zeiss) equipped with a 488 nm and 555 nm laser, a Plan-Neofluar 40x/1.30 oil immersion objective (Carl Zeiss) and appropriate emission filters. Stacks of 24.7 μm with a step size of 0.48 μm were acquired. Deconvolution was carried out using ImageJ 1.48. Point-spread-functions (PSF) were generated using the Diffraction PSF 3D plugin (OptiNav). Subsequently, deconvolution was carried out using the Deconvolution Lab plugin, Richardson-Lucy algorithm with 10 iterations (Sage et al., 2017). For each channel, a mask was generated from the binarized signal. To do so, first the contrast of the images was enhanced, a Median 3D filter was applied, and the image was thresholded. This was first performed for the tracer channel, and the resulting mask was copied to the second channel. Using the Erode (3D) function, the mask of the second channel was generated.

For visualization of ChR2-EYFP expression, images were taken from acute slices directly or they were first transferred into 4% PFA overnight, rinsed 3x with PBS, and mounted using anti-fading medium. They were visualized with an Axioscope 2 fluorescent microscope (Carl Zeiss) using 2.5x dry objective (Fluar, 0.12 NA). A Kiralux camera (Thorlabs GmbH) attached to the microscope with the corresponding program (ThorCam Version 3.5.1/ Micromanager Version 2.0) allowed taking images. A HXP 120 lamp (Carl Zeiss) served as the light source with appropriate filters (YFP: excitation: 450–490 nm, emission: 515–543 nm; tdTomato: excitation: 540–552 nm, emission: > 590 nm). Intensities of the two channels were adjusted offline due to the high tdTomato signal.

### 2.10 Data analysis and statistics

Raw traces were analyzed for IPSC amplitudes using a customized plugin for IGOR Pro 6 (Wavemetrics) or custom-written Matlab (Mathworks) code using the HEKA Patchmaster Importer (Keine, 2019). Mean data are shown with the S.E.M. In stepwise-increased stimulation experiments, IPSC amplitudes of single neurons were clustered using the K-means algorithm with squared Euclidean distance using Matlab (MacQueen, 1967). Silhouette plots were generated for each neuron to determine the optimal number of clusters (Kim and Kandler, 2003). Statistical analysis was performed using WinSTAT (R. Fitch Software). If data were normally distributed (Kolmogorov–Smirnov test), two-tailed paired t-tests were performed. Bonferroni correction was carried out for multiple comparisons. In these cases, the error probability (p) is indicated as *: *p* < 0.025, **: *p* < 0.005, or ***: *p* < 0.0005. For light-evoked IPSCs, the latency was determined as the time point after stimulation onset at which 10% of the peak amplitude was reached.

## 3 Results

**Figure 1:**
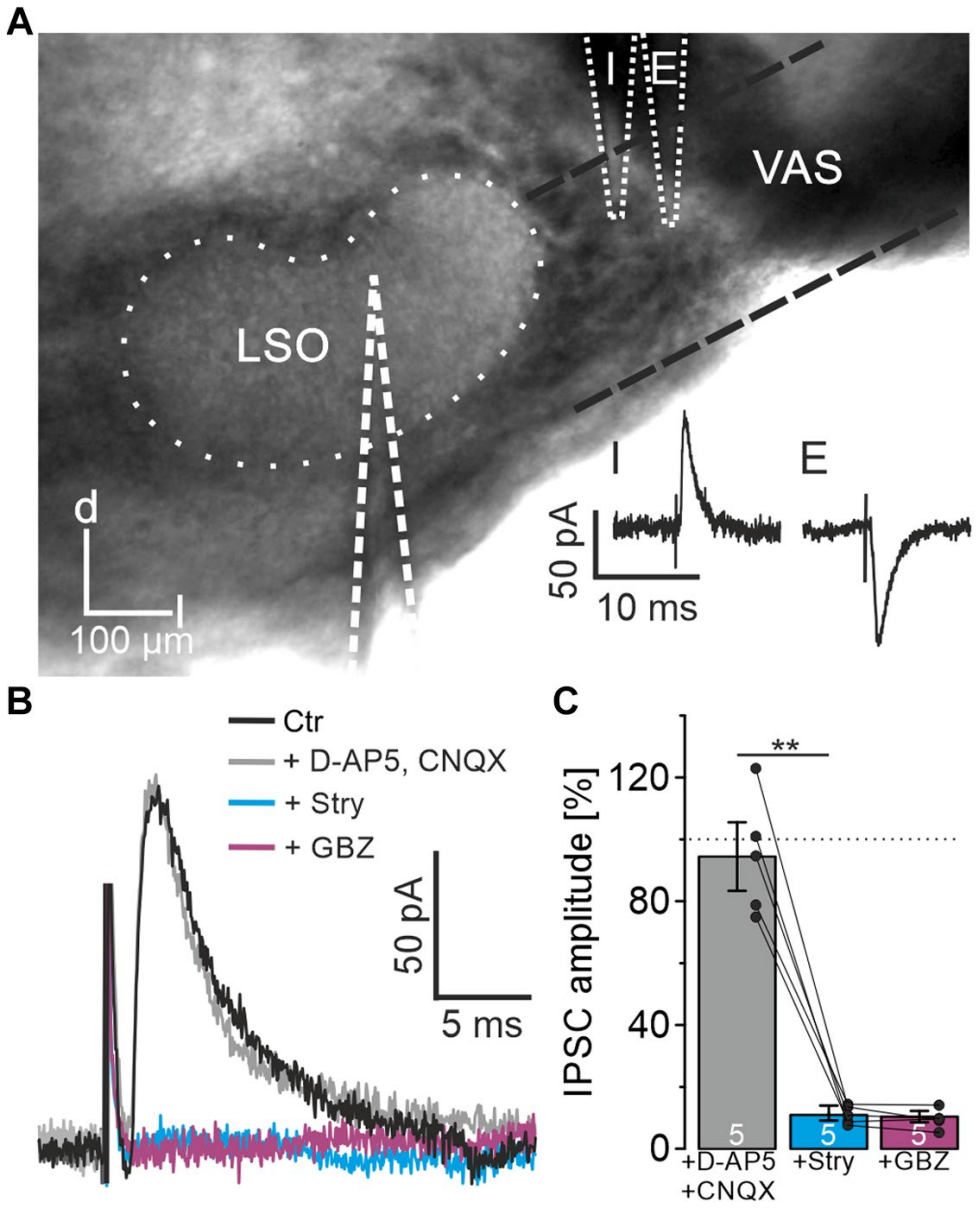
LSO principal neurons receive inhibitory glycinergic input originating from the ipsilateral VAS. **(A)** Stimulation in the VAS at slightly different positions results in inhibitory (I) or excitatory (E) PSCs. d = dorsal, l = lateral. **(B)** IPSCs after successive wash-in of different receptor antagonists. **(C)** Mean peak amplitudes in the consecutive antagonist situations relative to control (100%). Dots indicate values of single experiments (mean of 10 IPSCs each). Dotted line indicates amplitude before drug application. Situations were compared using a paired two-tailed t-test. Ctr = control, Stry = strychnine, GBZ = GABAzine.

When investigating ipsilateral synaptic inputs onto mouse LSO neurons, Wu and Kelly (1994) detected mixed excitatory and inhibitory postsynaptic responses in a subset of experiments. To investigate the synaptic composition of the projection in P10-12 mice in detail, we placed a stimulation electrode lateral to the LSO onto the VAS, roughly at the position of the facial nerve. In experiments on 37 LSO principal neurons, electrical stimulation resulted in inward currents, indicating activated excitatory projections most likely originating from the CN. However, in 8 neurons, outward currents were observed, indicating inhibitory synapses. In another 15 neurons, the response identified as an excitatory postsynaptic current could be converted into an IPSC by carefully repositioning the stimulation electrode by ∼10–50 μm (Figure 1A). These results confirmed that projections entering the LSO from the ipsilateral side are not purely excitatory but contain an inhibitory component. The finding prompted a further detailed investigation.

For pharmacological characterization of the IPSCs, several receptor blockers were washed-in under minimal stimulation conditions (Materials and Methods for details; *n* = 5, Figures 1B,C). In the presence of CNQX and D-AP5, IPSC amplitudes did not change relative to the control (94.4 ± 11.1%, *p* = 0.55). Further application of 0.5 μM strychnine, a concentration appropriate to prevent unspecific interactions with GABA receptors (Jonas et al., 1998), resulted in a strong decrease down to the noise level (11.0 ± 2.4%, *p* = 0.0006). Subsequent wash-in of GABAzine to assess a GABAergic contribution to the IPSCs showed no further reduction (10.4 ± 1.8%, *p* = 0.6). Therefore, the IPSCs could be pinned down as glycinergic. Since blockade of glutamatergic transmission did not affect IPSCs, the inputs were verified as monosynaptic.

**Figure 2:**
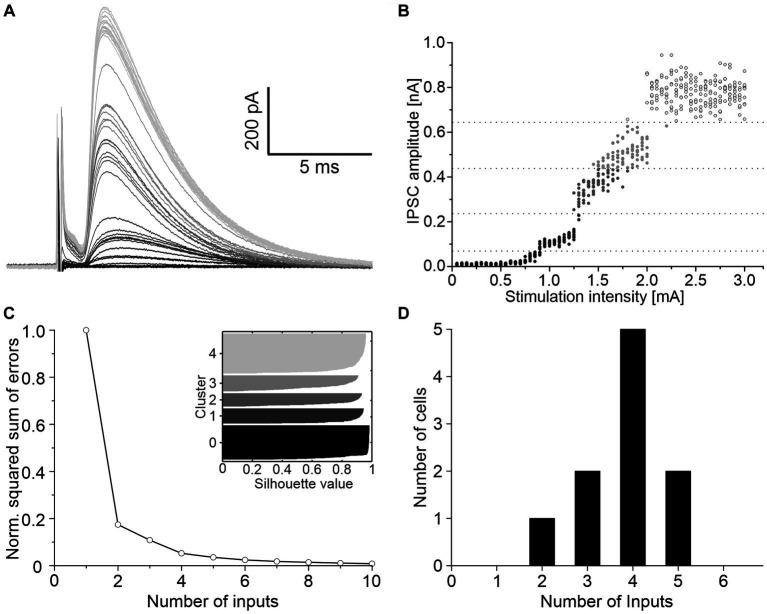
About four ipsilateral inhibitory neurons converge on a given LSO principal neuron. **(A)** Mean of 10 IPSCs evoked with increasing stimulation intensities of a representative neuron. Shading depicts the clustering of amplitudes, as shown in **C**. **(B)** All IPSC amplitudes of this neuron relative to their evoking stimulation intensities. Shades of grey, separated by dotted lines, depict the clustering of amplitudes. **(C)** Relative squared sum of errors of these IPSC amplitudes after K-means segmentation. Via silhouette plots the optimal number of clusters was determined as 4 plus 1 baseline cluster (inset). **(D)** Number of inhibitory inputs determined with K-means and silhouette plots for 10 neurons (arithmetic mean ± S.D.: 3.8 ± 0.9).

To determine the convergence ratio of ipsilateral inhibitory inputs onto a single LSO neuron, stimulation intensities were increased under blockage of excitatory neurotransmission. Successively larger electrical fields were thereby produced, and additional VAS axons were recruited, resulting in stepwise increasing IPSC amplitudes. The scenario is shown for a representative neuron in Figure 2A. The peak amplitudes of such IPSCs were analyzed with K-means clustering to separate the inputs into clusters, and silhouette plots were generated to determine the optimal number of clusters (Figures 2B,C). Across all neurons, the arithmetic mean of convergent inputs was about 4 (3.8 ± 0.3, Figure 2D).

**Figure 3:**
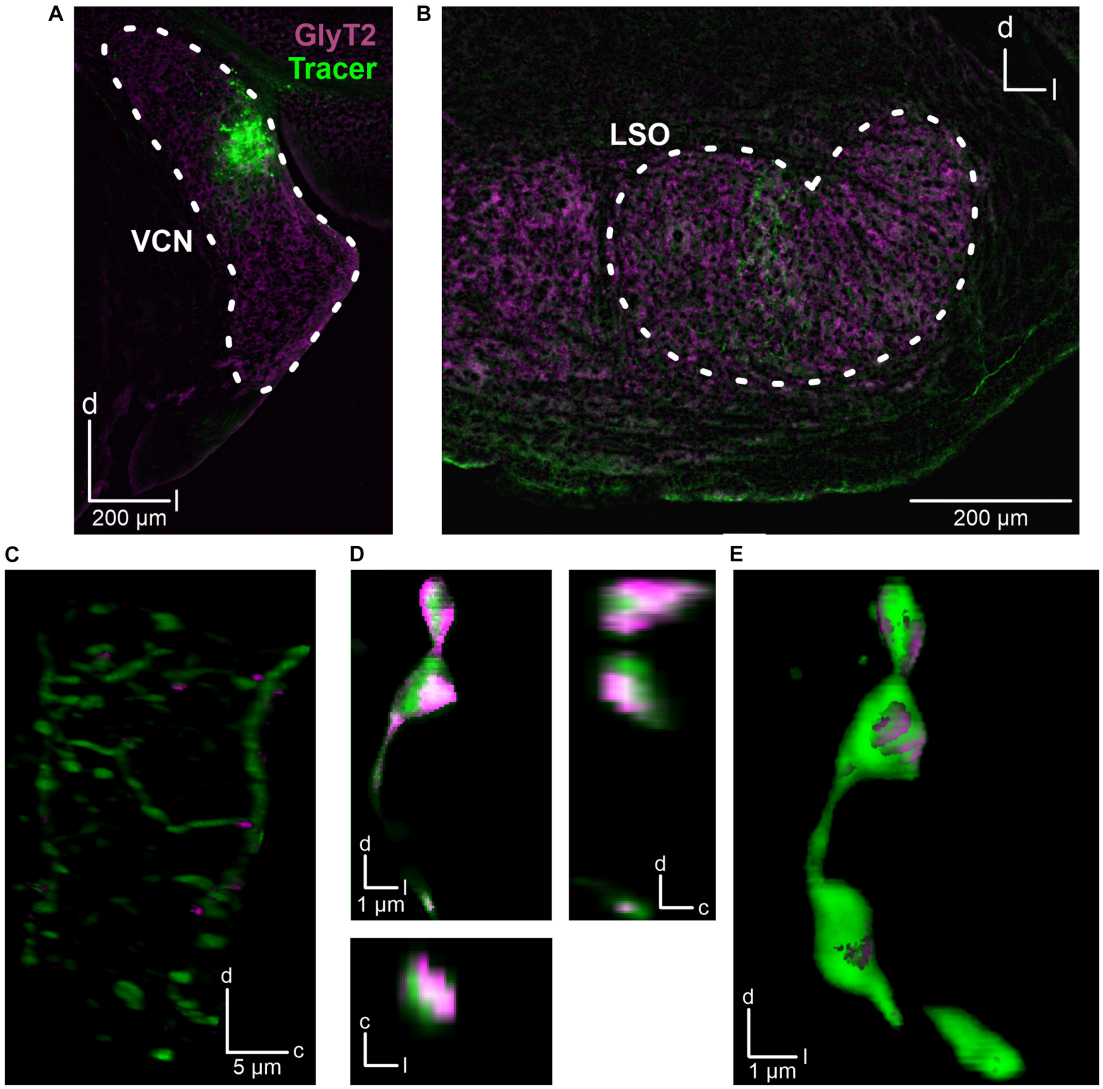
Anatomical tracing of inhibitory CN-LSO projections. **(A)** Injection site in the CN, visualized by the Cy3 signal of the dextran tracer (green). GlyT2 signals (magenta) were obtained via immunohistochemistry. **(B)** Signal of anterogradely transported dextran in the LSO. **(C)** Higher magnification of dextran and GlyT2 signals in the LSO following 3D reconstruction. The image is a Z-projection across 21 μm. Only GlyT2 signal overlapping with tracer is displayed. **(D)** Cy3- and GlyT2-positive terminals in the LSO (different animal than in **A–C**). d = dorsal, l = lateral, c = caudal. **(E)** 3D reconstruction of the axon terminals shown in **D**.

The fact that the CN is the main ipsilateral input to the LSO (Cant and Benson, 2003) and that we had placed the stimulation electrode close to the VAS, which carries ascending axons originating from the CN, suggests that the observed inhibitory input also originates from CN neurons. To address this hypothesis, we performed anterograde tract tracing experiments and injected dextran tracer into the CN (three adult mice). For histological and morphological analysis, slices of the CN and SOC were prepared and counterstained for GlyT2, a well-established marker of glycinergic axon terminals (Poyatos et al., 1997). The axonal tracer was visualized using Cy3. Injection sites in the CN were verified (Figure 3A) and tracer signal was detected in the LSO (Figures 3B,C). Following deconvolution of confocal images, we found co-distribution of tracer and GlyT2 signals. 3D reconstructions clearly displayed glycinergic presynaptic boutons (Figures 3D,E).

**Figure 4:**
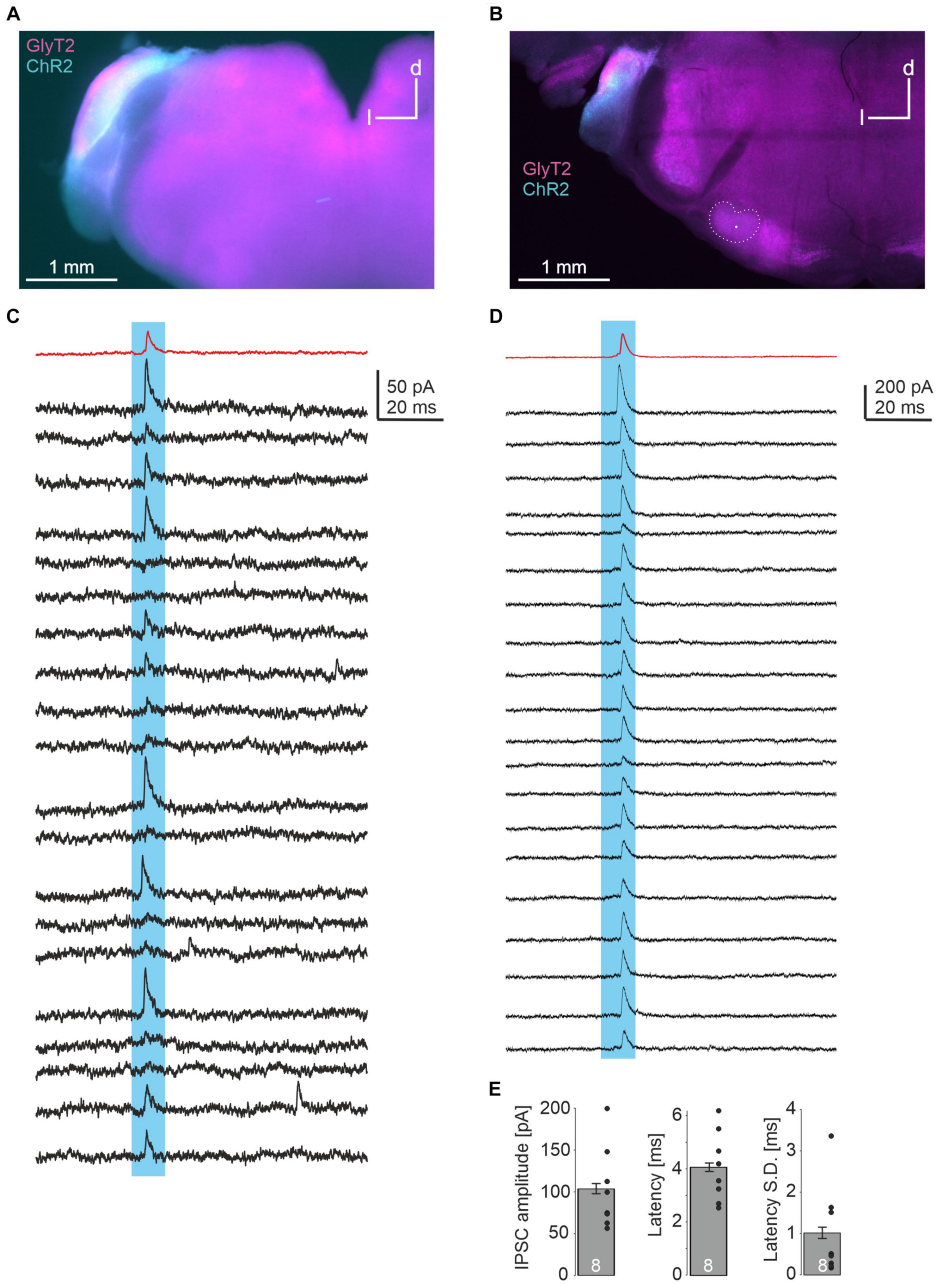
Optogenetic identification of inhibitory CN-LSO input. **(A)** Visualization of ChR2 expression in the CN in an acute slice via EYFP signal after injection of AAV. d = dorsal, l = lateral **(B)** Same as in **A**, but image taken from a PFA-fixated, mounted slice. The LSO is outlined and the recording site is marked by a dot. **(C,D)** Examples of IPSCs in LSO neurons (corresponding to the slices shown in **A,B**) evoked by blue light pulses. Twenty responses are shown in black traces, and the average trace is in red. **(E)** Statistical summary of peak amplitudes, latency from light stimulation onset (both averaged across 20 repetitions for each experiment, excluding failures), and jitter of IPSC onset across 20 repetitions for all 8 neurons displaying light-evoked IPSCs. Dots represent values of single experiments. S.D. = standard deviation.

In a last series of experiments, we injected a double-floxed ChR2-EYFP construct via AAV into the CN of GlyT2-Cre/tdTomato^flox^ mice. These animals express Cre under the GlyT2 promotor as well as tdTomato through cross-breeding with tdTomato^flox^ mice. 18–20 days later, acute brainstem slices were prepared. EYFP signal within the injected CN overlapped with TdTomato, indicating the expression of ChR2 in glycinergic CN neurons (Figures 4A,B). LSO principal neurons were recorded and putative glycinergic axons and presynapses were stimulated by a 470 nm LED focused onto the slice through the objective. In 8 of 18 neurons (2 of 8 from one slice in one animal, 6 of 10 from 3 slices in another), light-evoked IPSCs were observed (Figures 4C,D), with peak amplitudes of 104 ± 6 pA. Response latencies were quite short (4.1 ± 0.2 ms), with small onset jitter of the signals (standard deviation of latency of 1.0 ± 0.1 ms across 20 repetitions; Figure 4E). From these data we conclude that LSO neurons receive inhibitory glycinergic input from the ipsilateral CN.

## 4 Discussion

In the present study, we characterize an inhibitory, glycinergic projection from the ipsilateral CN to LSO principal neurons in mice. We do so by employing anatomical tracing, patch-clamp recordings in acute slices, and optogenetics. While past studies had suggested the existence of such a pathway (Brownell et al., 1979; Glendenning et al., 1991; Wu and Kelly, 1994), the origin has so far remained unproven.

Besides the main excitatory projection from the ipsilateral CN and inhibitory input from the MNTB, LSO neurons receive synaptic input from other sources, such as descending input from the neocortex (Feliciano et al., 1995). Further inhibitory input originates from the contralateral ventral nucleus of the trapezoid body (Warr and Beck, 1996), and in bats from the ipsilateral lateral nucleus of the trapezoid body (LNTB; Kuwabara and Zook, 1992). However, projections from the LNTB to the LSO were not observed in rodents (Gómez-Álvarez and Saldaña, 2016). Amongst these inputs, the inhibitory input from the CN, characterized in the present study, may be in a particularly good position to affect firing rates of LSO neurons upon sound stimulation, as it is part of the early ascending pathway and may thus be highly synchronous with excitatory CN input. With merely ~4 axons converging on a single LSO principal neuron, the inhibitory input is strongly outnumbered by the excitatory input from the CN, which averages 8–20 neurons (Noh et al., 2010; Gjoni et al., 2018). While these numbers do not offer a direct value of synaptic strength, they may serve as an estimate of their physiological relevance. A broad range of the average number of MNTB axons (4–10) converging on one LSO neuron has been reported (Noh et al., 2010; Walcher et al., 2011; Hirtz et al., 2012; Clause et al., 2014; Müller et al., 2019), hampering comparison to the inhibitory CN-LSO projection at this point.

It should be noted that in past studies, which used exclusively electrical stimulation of axons, IPSCs in LSO neurons might be attributed to activated glycinergic neurons/axons originating from sources other than the CN. Our approach of optogenetically activating axons originating from glycinergic CN neurons dissipates these concerns. Furthermore, the fact that the optogenetic approach, in contrast to electrical stimulation, allows to specifically and exclusively activate glycinergic neurons corroborates a monosynaptic nature of the investigated synaptic connection. Short response latencies with minimal jitter strengthen this conclusion and speak against the possible concern that unspecific expression of ChR2 in non-glycinergic, excitatory CN neurons might have led to polysynaptic light-evoked IPSCs by indirectly stimulating other glycinergic sources, such as LNTB neurons. This is also unlikely because we never observed light-evoked excitatory postsynaptic currents in LSO neurons, which would be expected in case of substantial expression of ChR2 in excitatory CN neurons. Furthermore, as discussed above, whether the LNTB projects to the LSO in rodents is disputed. Nevertheless, a caveat that should be considered is that our results regarding electrically evoked IPSCs might still include a proportion of sources other than the CN.

What may be the function of the inhibitory ipsilateral CN-LSO projection? Inhibitory sidebands are present at many auditory centers, including CN (Ding and Voigt, 1997), inferior colliculus (Xie et al., 2007; Grécová et al., 2009; Pollak et al., 2011), and cortex (O'Connell et al., 2011; Ji and Patrick, 2021). In these regions, they increase stimulus selectivity. Inhibitory sidebands in response to ipsilateral stimulation have also been observed in the cat LSO (Brownell et al., 1979), and there is also evidence for them in gerbils (Franken et al., 2018). The here described inhibitory connection from the ipsilateral CN is a good candidate mediating this sideband inhibition. These considerations highlight quite complex neuronal computation already at early auditory brainstem stations. As they are at this point mostly speculatory, they need to be addressed in future studies.

An open question from our work is which CN neuron type provides the inhibitory connection to the ipsilateral LSO. Our axonal tract tracing studies show the ventral cochlear nucleus (VCN) as a source. While the dorsal cochlear nucleus cannot be fully ruled out as a further source, given that expression of ChR2 was observed in it in one of our optogenetic experiments (Figure 4A), projections from the dorsal cochlear nucleus to the LSO have not been observed to our knowledge (discussed in Romero and Trussell, 2022). Spherical bushy cells in the VCN comprise the main output neurons to the LSO (Cant and Benson, 2003), yet no report exists that some of them are inhibitory. T-stellate cells, which mainly project via the trapezoid body, also project to the LSO (Doucet and Ryugo, 2003). However, these neurons appear to be purely excitatory (Fujiyama et al., 2009). D-stellate cells as well as the recently discovered L-stellate cells are the only known inhibitory neuron types in the VCN (Ngodup et al., 2020). Interestingly, L-stellate cells are ~10-fold more numerous than D-stellate cells (3,250 vs. 380). Both subtypes are glycinergic, but at present, there is no evidence that they project into the LSO (NB: D stands for the dorsalward trajectory, Ferragamo et al., 1998, L stands for the Local projections, Ngodup et al., 2020). Nevertheless, these observations carefully hint at a subclass of stellate neurons being the potential source of the projection analyzed in the present study. Further research needs to be conducted to address this question.

In conclusion, our results about a direct inhibitory glycinergic projection from the CN to the ipsilateral LSO further challenge the classical idea that this major ascending pathway is purely excitatory. They also contribute to the increasing awareness about the complexity of synaptic projection patterns and acoustic information processing at early stations in the mammalian brainstem.

## Data availability statement

The raw data supporting the conclusions of this article will be made available by the authors, without undue reservation.

## Ethics statement

The animal study was approved by Landesuntersuchungsamt Rheinland-Pfalz. The study was conducted in accordance with the local legislation and institutional requirements.

## Author contributions

DW: Data curation, Formal analysis, Investigation, Visualization, Writing – review & editing. ES: Data curation, Formal analysis, Investigation, Visualization, Writing – review & editing. JW: Data curation, Formal analysis, Investigation, Writing – review & editing. NP-K: Investigation, Writing – review & editing. AF: Data curation, Formal analysis, Software, Visualization, Writing – review & editing. SW: Investigation, Writing – review & editing. EF: Conceptualization, Project administration, Resources, Supervision, Writing – review & editing. JH: Conceptualization, Methodology, Project administration, Resources, Supervision, Visualization, Writing – original draft, Writing – review & editing.
